# Neural Differentiation Is Inhibited through HIF1*α*/*β*-Catenin Signaling in Embryoid Bodies

**DOI:** 10.1155/2017/8715798

**Published:** 2017-12-20

**Authors:** Josef Večeřa, Jana Kudová, Jan Kučera, Lukáš Kubala, Jiří Pacherník

**Affiliations:** ^1^Department of Experimental Biology, Faculty of Science, Masaryk University, 62500 Brno, Czech Republic; ^2^Institute of Biophysics, Academy of Sciences of the Czech Republic, 61265 Brno, Czech Republic; ^3^International Clinical Research Center, St. Anne's University Hospital, 65691 Brno, Czech Republic

## Abstract

Extensive research in the field of stem cells and developmental biology has revealed evidence of the role of hypoxia as an important factor regulating self-renewal and differentiation. However, comprehensive information about the exact hypoxia-mediated regulatory mechanism of stem cell fate during early embryonic development is still missing. Using a model of embryoid bodies (EBs) derived from murine embryonic stem cells (ESC), we here tried to encrypt the role of hypoxia-inducible factor 1*α* (HIF1*α*) in neural fate during spontaneous differentiation. EBs derived from ESC with the ablated gene for HIF1*α* had abnormally increased neuronal characteristics during differentiation. An increased neural phenotype in *Hif1α^−/−^* EBs was accompanied by the disruption of *β*-catenin signaling together with the increased cytoplasmic degradation of *β*-catenin. The knock-in of *Hif1α*, as well as *β*-catenin ectopic overexpression in *Hif1α^−/−^* EBs, induced a reduction in neural markers to the levels observed in wild-type EBs. Interestingly, direct interaction between HIF1*α* and *β*-catenin was demonstrated by immunoprecipitation analysis of the nuclear fraction of wild-type EBs. Together, these results emphasize the regulatory role of HIF1*α* in *β*-catenin stabilization during spontaneous differentiation, which seems to be a crucial mechanism for the natural inhibition of premature neural differentiation.

## 1. Introduction

During early mammalian development, embryonic cells are localized into the microenvironment of the uterus, where the embryo is able to survive and grow in hypoxia (low oxygen tension) before the functional vascular system is formed and oxygen and nutrition are supplied to embryonic tissue [1]. The impact of hypoxia (ranging from 21.6 to 36 mm Hg, 3–5% O_2_) on normal early embryonic development has been emphasized by several research groups [2, 3]. Intriguingly, most embryonic and adult stem cell populations, including hematopoietic, mesenchymal, and neural stem cells, are naturally present in hypoxic conditions and oxygen level plays a crucial role in the determination of stem cell fate [4–6]. Besides the oxygen gradient, developmental morphogens including ligands of the Wnt family have a relevant impact on the temporal and spatial induction as well as specification of all germ layers [7–10]. However, the effect of low oxygen tension and morphogens on stem cell fate remains controversial, especially in the context of ESC self-renewal and differentiation.

Cellular responses to hypoxia are mainly mediated via hypoxia-inducible factors (HIF). These transcription factors are master regulators of adaptation processes in response to decreased oxygen supply, including angiogenesis, glycolysis, and red blood cell production [11]. Besides their role in the regulation of metabolic and angiogenic responses, HIFs are involved in the control of the proliferation and differentiation of most stem cell populations [12]. Importantly, the specific ablation of the HIF1*α* isoform during ESC differentiation is associated with impaired vasculogenesis and cardiomyogenesis [6, 13]. A regulatory role for HIF1*α* in stem cell fate and neural differentiation has been suggested, mostly through a direct interaction between HIF1*α* and regulatory elements of the crucial developmental pathways activated by Wnt or Notch ligands [14, 15]. However, there are still question marks about the exact role of HIF1*α* and its interaction with other signaling pathways during stem cell differentiation, especially in early neural differentiation.

In this study, we highlight the role of HIF1*α* in early neural differentiation using a model of EBs derived from murine ESC. In the EB microenvironment, ESC undergo spontaneous differentiation into all three germ layers and recapitulate early mammalian development [7, 16]. Our results show that the stabilization of HIF1*α* in the EB microenvironment appears to be a crucial regulatory mechanism for the inhibition of premature neural differentiation. We also address the crosstalk between HIF1*α* and *β*-catenin signaling and evaluate the impact of this interaction on neural fate in ESC differentiation.

## 2. Materials and Methods

### 2.1. ESC Cultivation and Differentiation

mESC (R1 cell line; *Hif1α^−/−^* cells derived from R1 were kindly provided by Peter F. Carmeliet; Vesalius Research Center, VIB, University of Leuven, 3000 Leuven, Belgium) were cultivated on gelatin-coated dishes in Dulbecco's modified Eagle's medium (DMEM; HyClone; Logan, UT, USA) supplemented with 15% fetal bovine serum (Gibco; Carlsbad, CA, USA), 100 IU/ml penicillin, and 0.1 mg/ml streptomycin (Sigma-Aldrich; St. Louis, MO, USA), 1x nonessential amino acid (Gibco; Carlsbad, CA, USA), 0.05 mM *β*-mercaptoethanol (Fluka; Buchs, Switzerland), and 1000 U/ml of leukemia inhibitory factor (Chemicon; Temecula, CA, USA). ESC were differentiated as described previously [13]. Briefly, a suspension of ESC (2.5 × 10^6^ cells/ml) was seeded on the surface of silicone mold-made microwells in 1.5% agarose (VWR) to form embryoid bodies (EBs). After 24 hours of aggregate formation (day 0), the EBs were transferred onto an agar-coated dish and cultivated without leukemia inhibitory factor supplementation. All transfection procedures were performed on EBs at this point, prior to their transfer to agar plates. For neural differentiation, 5-day-old EBs (5d) were seeded on gelatin-coated dishes in DMEM/F-12 (1 : 1) medium (Gibco; Carlsbad, CA, USA) supplemented with insulin-transferrin-selenium (Gibco) and antibiotics (specification above) and the EBs were grown for 10 days (5 + 10d) in adherent culture.

### 2.2. Quantitative Real-Time PCR

Total RNA was extracted from ESC and EBs using the UltraClean Tissue & Cells RNA Isolation Kit (MO BIO Laboratories; Carlsbad, CA, USA). 0.5 *μ*g of total RNA was used for cDNA synthesis with the Mu-MLV reverse transcriptase kit and Oligo(dT) primers according to the manufacturer's instructions (Thermo Fisher Scientific Inc., USA). qPCR reactions were performed in a LightCycler480 instrument using a LightCycler®480 DNA SYBR Green I Master (Roche, Switzerland). The program steps were described previously [17]. Data were normalized to glyceraldehyde-3-phosphate dehydrogenase (GAPDH) mRNA expression and presented as 2^−∆cq^. The sequences of primers used for individual markers were as follows:

*Nestin*:

F-CATACAGGACTCTGCTGGAGG, R-GAGAAGGATGTTGGGCTGAG;

*Ascl1/Mash1*:

F-GGTCTCGTCCTACTCCTCCG, R-GCTGCCATCCTGCTTCCAAA;

*Hif1α*:

F-TTCTGGATGCCGGTGGTCTA, R-AAACCATGTCGCCGTCATCT;

*Tcf1/Tcf7*:

F-CAGCTCCCCCATACTGTGAG, R-TGCTGTCTATATCCGCAGGAA;

*Lef1*:

F-TCCTGAAATCCCCACCTTC, R-ACCCGTGATGGGATAAACAG;

*Tcf3/Tcf7like1*:

F-CTGAGCAGCCCGTACCTCT, R-AGGGGCCATTTCATCTGTAG;

*Tcf4*:

F-ATTTGTGGCCATTGAAGGTT, R-GTCCCTAAGGCAGCCATTC;

*Axin2*:

F-GCGCTTTGATAAGGTCCTGG, R-TCATGTGAGCCTCCTCTCTTTT;

*NeuroD1*:

F-AACCTTTTAACAACAGGAAGTGGA, R-CTCATCTGTCCAGCTTGGGG;

*Gapdh*:

F-AAGGGCTCATGACCACAGTC, R-CATACTTGGCAGGTTTCTCCA.

### 2.3. Western Blotting, Immunoprecipitation, and Immunohistochemistry

Cells were lysed using SDS buffer (50 mM Tris-HCl, 100 mM NaCl, 10% glycerol, 1% SDS, 1 mM EDTA, and protease and phosphatase inhibitors (Roche; Basel, Switzerland) at pH 7.4) at particular time intervals (ESC, 5d, 5 + 5d, and 5 + 10d). Proteins were separated using an 8% or 10% SDS-PAGE gel, then transferred onto a polyvinyl difluoride membrane (Merck Millipore; Darmstadt, Germany), and blocked in 5% low-fat milk in TBS-T buffer (Tris, 0.05% Tween20). The detection of selected proteins was then performed using the following specific primary antibodies: mouse monoclonal anti-TUJ1 (Sigma-Aldrich, T5076), mouse monoclonal anti-PAX6 (DSHB, University of Iowa, deposited by Kawakami A.), rabbit polyclonal anti-BLBP (Abcam, 27171), rabbit polyclonal anti-HIF1*α* (Genetex, 127309), mouse monoclonal anti-vinculin (Sigma-Aldrich, V9264), mouse monoclonal anti-*β*-catenin (BD, 610153), rabbit polyclonal anti-phospho-*β*-catenin (CS, number 9561), goat polyclonal anti-*β*-actin (Santa Cruz), goat polyclonal anti-laminB (Santa Cruz), rabbit monoclonal anti-AKT (CS, number 4691), and mouse monoclonal anti-LewisX/Forse1 (DSHB, University of Iowa, deposited by Patterson P.H.). The following secondary antibodies (all from Sigma-Aldrich, all anti IgG) conjugated with peroxidase were used: goat anti-mouse (A4416), goat anti-rabbit (A0545), and rabbit anti-goat (A4174). The protein levels of neural markers were normalized to the vinculin signal; the level of phosphorylated *β*-catenin was normalized to the total *β*-catenin signal. The optical densities of protein bands were quantified by ImageJ software (https://imagej.net/Contributors, NIH, USA) using the Gel analysis feature, and extrapolated values from the peak area were subjected to statistical analysis.

For immunoprecipitation analysis, we used a modified protocol employed by the research group of Dr. Bryja. Tris buffer (pH 7.4; 50 mM TRIS, 150 mM NaCl, 1 mM EDTA, 0.5% NP-40, 5% glycerol, 1 mM DTT, and protease inhibitor cocktail (Roche; Basel, Switzerland)) was used for gentle cell lysis and protein complex preservation. Two-day-old EBs were lysed for 45 minutes on ice, mixed, and centrifuged for 10 minutes at 1500*g* to separate the cytoplasmic fraction in the supernatant. The resulting pellet was dissolved in lysis buffer, sonicated for 6 s (low intensity), and centrifuged for 10 minutes to separate the nuclear protein fraction in the supernatant. Fractions with antigen samples were incubated with anti-HIF1*α* antibody (or rabbit polyclonal IgG for non-specific binding control, Cell Signaling number 3900), added to washed magnetic beads covered with A/G protein mixture, and incubated overnight at 4°C. Next day, all samples were washed thoroughly to remove the unbound protein fraction, the beads were boiled for 5 minutes in Laemmli buffer, and the denaturated samples were analyzed by western blot. Each fraction was sampled prior to the addition of magnetic beads for subsequent WB analysis and total protein estimation.

Prior to immunohistochemistry analysis, cells/EB colonies attached to glass surface were washed 2 times in PBS, fixed in 4% (wt/wt) paraformaldehyde (PFA) for 15 min/4°C, again washed in PBS, and incubated with 1% BSA/0.3% Triton X for 1 hr/RT. After blocking reaction, primary antibodies were dissolved in 1% BSA/0.3% Triton X at 4°C overnight. Next day, samples were washed 3 times in PBS and incubated with secondary antibodies for 1 hr/RT. After this, samples were washed 3 times in PBS and samples were mounted using VectaShield H1000 (Vector Laboratories, Burlingame). Immunofluorescence was visualized with an Olympus CLSM confocal microscope. Primary antibody against TUJ1 was the same as for western blotting; secondary antibody was Alexa Fluor 568 donkey anti-rabbit.

### 2.4. Cell Transfections and Luciferase Reporter Assay

EBs were transfected on day 0 using polyethyleneimine (PEI, Sigma-Aldrich, 408727). The following plasmid DNAs were used (all at a concentration of 0.8 *μ*g/well of a 12-well plate), either separately or by cotransfection, if desired: plasmid expressing nondegradable *β*-catenin mutated on S33 (Professor H.R. Korswagen, Utrecht University, Netherlands), plasmid overexpressing mouse *Hif1α* (Professor Poelinger Lab, Karolinska Institutet, Sweden), and empty pcDNA3 plasmid for control transfections. Luciferase reporter assay was used to quantify the *β*-catenin-mediated transcriptional activation of TCF/LEF binding sites in 2-day-old EBs. Aggregates cultivated in agar microwells (12-well plate) were transfected on day 0 with 0.8 *μ*g of Super8XTOPflash/Super8XFOPflash luciferase plasmid (Professor R.T. Moon, University of Washington, USA) together with 0.4 *μ*g Renilla luciferase reporter plasmid (Promega, Madison, WI, USA). 48 hours after transfection, reporter activity was measured using the Dual-Luciferase Reporter Assay System Kit (Promega). Analyses of each experimental condition were performed in triplicates. The luciferase activity of SuperTOP/FOPflash was normalized to the Renilla luciferase signal.

### 2.5. Statistical Analysis

The results were subjected to statistical analyses (Student's *t*-test; one-way ANOVA with Tukey's multiple comparison posttest), and statistically significant differences were defined as follows: *p* < .05 (^∗^), *p* < .01 (^∗∗^), and *p* < .001 (^∗∗∗^). Results are presented as mean + SEM.

## 3. Results

### 3.1. *Hif1α* Deficiency Is Associated with Abnormal Neurogenesis *In Vitro*

**Figure 1:**
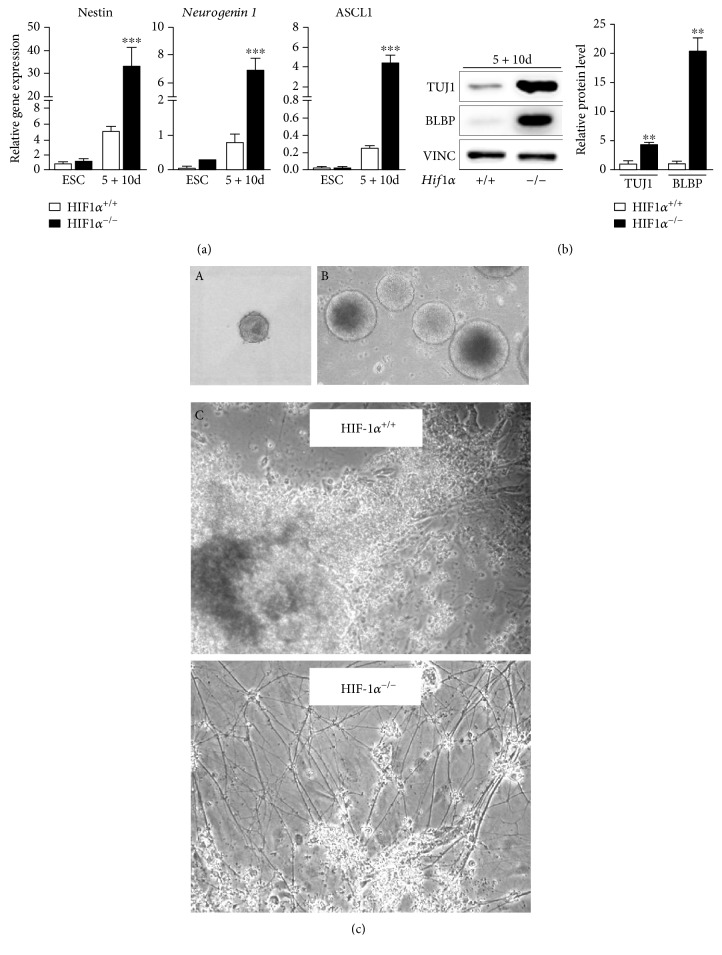
*Hif1α* deficiency is associated with abnormal neurogenesis *in vitro*. (a) Relative gene expression of selected markers *Nestin*, *Neurogenin 1*, and *Ascl1* (achaete-scute complex-like 1) involved in neurogenesis detected at the mRNA level during the differentiation of wild-type and *HIF1α*-deficient ESC by real-time quantitative PCR analysis in undifferentiated ESC (ESC) and in EBs differentiated for 10 days in adherent culture (5 + 10d). Data are presented as means ± SEM from at least 5 independent experiments (one-way ANOVA for each set of ESC and 5 + 10d; significance indicated only for dif.*Hif1α*^+/+^ versus dif.*Hif1α*^−/−^). (b, A) Protein levels of TUJ1 and BLBP (brain lipid-binding protein) detected by western blot in EBs differentiated for 10 days in adherent culture (5 + 10d). (b, B) Densitometric analysis was performed on the basis of 3 independent experiments (*t*-test for each marker separately, *Hfi1α*^+/+^ versus *Hif1α*^−/−^). (c, A) Illustrative picture of aggregate grown for 24 hrs (0d) in agarose microwell and (c, B) of 5-day-old EBs (5d) grown on an agar-coated plate. Scale bar: 200 *μ*m. (c, C) Light microscope images illustrating the morphology of *Hif1α*^+/+^ and *Hif1α*^−/−^ EBs after 10 days of differentiation in adherent culture (5 + 10d).

We first explored the developmental fate preferred by *Hif1α^−/−^* EBs during spontaneous differentiation. RT-qPCR analysis of EBs differentiated for 10 days in an adherent culture (5 + 10d) revealed the substantially increased expression of genes involved in early neurogenesis, such as *Nestin*, *Neurogenin 1*, and *ASCL1* (Figure 1(a)). A neural lineage shift in differentiated *Hif1α^−/−^* EBs was also confirmed at the protein level, showing a significant increase in the neuroectodermal marker BLBP and marker of immature neurons TUJ1 (Figure 1(b)). Immunohistochemical staining confirmed enhanced expression of TUJ1 within differentiated *Hif1α^−/−^* EB colonies (Supplementary Figure A). We also checked the morphology of differentiated EB colonies using a light microscope and observed many *Hif1α^−/−^* colonies sprouting multiple neurite projections from the periphery, in contrast to *wt* colonies, where we detected almost no neurites at all (Figure 1(c), C; Supplementary Figure B). These results emphasize the fact that EBs derived from ESC lacking the *Hif1α* gene generally prefer a neural lineage fate during spontaneous differentiation.

### 3.2. Stabilized HIF1*α* in EBs Prevents Neural Differentiation

**Figure 2:**
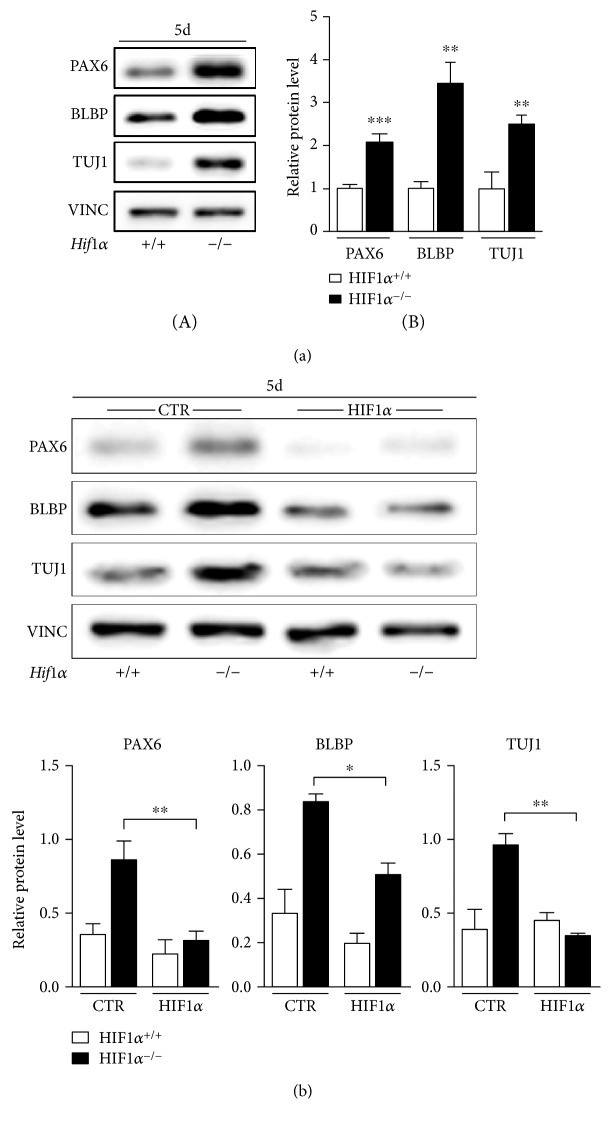
Stabilized HIF1*α* in EBs prevents neural differentiation. (a, A) Protein levels of neural markers PAX6, BLBP, and TUJ1 detected by western blot in 5-day-old EBs derived from wild-type and *Hif1α*-deficient ESC. (a, B) Densitometric analysis and quantification (*t-*test for each marker separately, *Hif1α*^+/+^ versus *Hif1α*^−/−^; *n* = 8 for PAX6, *n* = 5 for BLBP, and *n* = 4 for TUJ1). (b, A) PAX6, BLBP, and TUJ1 protein levels in 5-day-old EBs (5d) after transfection with empty plasmid (CTR) or plasmid overexpressing mouse *Hif1α* (HIF1*α*). (b, B) Densitometric analysis and quantification (one-way ANOVA; *n* = 7 for PAX6, *n* = 4 for BLBP, and *n* = 3 for TUJ1).

After one day of cellular aggregate formation (Figure 1(c), A), aggregates were shaped in spherical organoids and were grown for 5 days on agar-coated plates (Figure 1(c), B). We did not observe significant differences in morphology or size of *Hif1^−/−^* EBs compared to *wt* (Supplementary Figure C). Interestingly, we observed changes in the neural phenotype in 5-day-old *Hif1α^−/−^* EBs. Using western blot analysis, we found a significant increase in the transcription factor PAX6 in *Hif1α^−/−^* EBs, an important and specific regulator of early neurogenesis, and the same increasing trend was also shown by the neural markers BLBP and TUJ1 (Figure 2(a)). Importantly, the transient knock-in of *Hif1α* during the formation of *Hif1α^−/−^* EBs led to a significant decrease in PAX6, BLBP, and TUJ1 protein levels (Figure 2(b)). This clearly demonstrated that the absence of HIF1*α* caused the enrichment of the neuroectodermal/neuronal population during spontaneous differentiation and that the recruitment of HIF1*α* during this period was a sufficient impulse for blocking this abnormal phenotype. We additionally examined whether the increased neurogenesis observed in *Hif1α^−/−^* EBs could be a consequence of downregulated vascular endothelial growth factor (VEGF), a potent angiogenic and neurotrophic factor, and downstream target of HIF1*α* signaling [18, 19]. We found that VEGF added to EBs during differentiation for ten days (6–13d) partially lowered neurophenotype in *Hif1α^−/−^* EBs, especially the level of LewisX, a marker of neural stem/progenitor cells; however, we hardly saw decrease in neural markers in *Hif1α^−/−^* EBs when VEGF was added during the formation of EBs (Supplementary Figure D). This led us to the conviction that there must be another signaling pathway dependent on HIF1*α*, which accounts for the blockade of the neural phenotype during EB formation.

### 3.3. The Increased Neurophenotype in *Hif1α^−/−^* EBs Is Associated with Decreased Nuclear *β*-Catenin Activity

**Figure 3:**
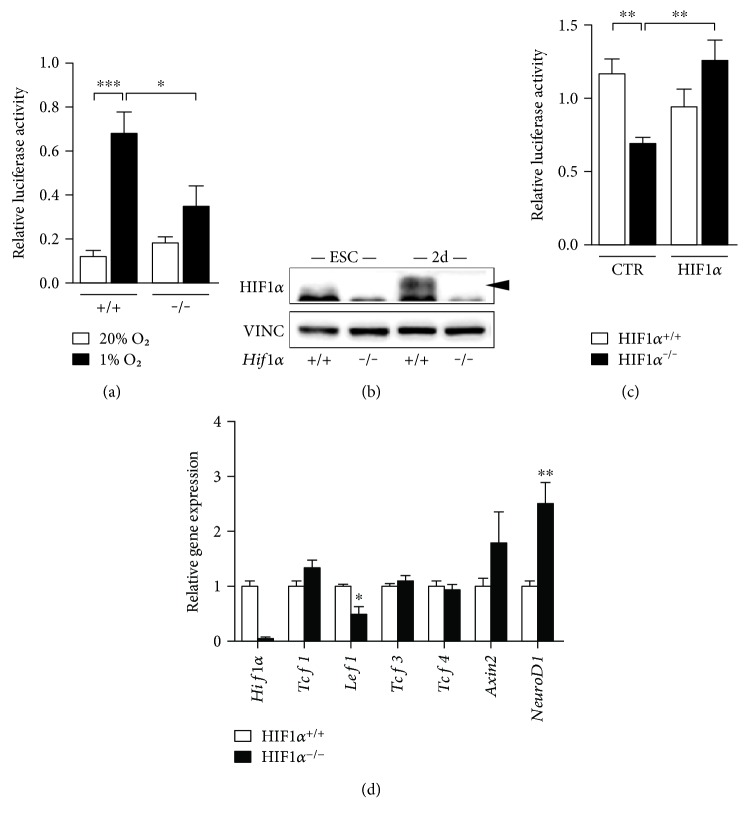
Increased neurophenotype in *Hif1α^−/−^* EBs is associated with decreased nuclear *β*-catenin activity. (a) TOPflash assay performed in 2-day-old EBs (2d) to compare *β*-catenin nuclear activity in wild-type (+/+) and *Hif1α*-deficient (−/−) ESC cultivated in 20% (white bars) and 1% (black bars) of oxygen. Quantification is based on at least 3 independent experiments (one-way ANOVA). (b) Western blot analysis (representative picture) showing stabilization of HIF1*α* protein in 2-day-old EBs compared to ESC. Black arrow indicates band of HIF1*α* at expected molecular weight. (c) TOPflash assay in 2-day-old EBs (2d) (white bars for wt, black bars for *Hif1α*^−/−^) after transfection with either empty plasmid (CTR) or plasmid overexpressing mouse *Hif1α* (HIF1*α*). Quantification is based on at least 7 independent experiments (one-way ANOVA). (d) Relative gene expressions of *Hif1α*, *Tcf1*, *Lef1*, *Tcf3*, *Tcf4*, *Axin2*, and *NeuroD1* detected in 2-day-old EBs derived from wild-type (white bars) and *Hif1α*-deficient cells (black bars) by real-time quantitative PCR analysis. Quantification is based on at least 3 independent experiments (*t*-test for each marker separately, *Hif1α*^+/+^ versus *Hif1α*^−/−^).

The involvement of the *β*-catenin pathway in spontaneous differentiation and early neurogenesis has already been evidenced by several authors (Aubert et al., [8]; Naito et al., [9]; ten Berge et al., [10]). However, a direct connection between hypoxia/HIF1*α* and *β*-catenin in this process has not yet been thoroughly investigated. We used TOP-FLASH luciferase assay to monitor the activity of nuclear *β*-catenin, which is based on the ability of *β*-catenin to activate several TCF/LEF binding regions on the luciferase gene promoter [20]. First, we monitored *β*-catenin activity under hypoxic conditions in ESC after transfection with TOP-FLASH plasmid. After 24 hours of growth in hypoxic conditions (1% O_2_), we observed significantly higher luciferase activity compared to ESC cultivated in normoxic conditions (Figure 3(a)); more importantly, the increase in luciferase activity was evidently dependent on the stabilization of HIF1*α* in hypoxia. To further investigate the connection between HIF1*α* and *β*-catenin during spontaneous differentiation on the endogenous level, we focused on the analysis of 2-day-old EBs (2d), where we clearly detected the stabilization of HIF1*α* protein, suggesting hypoxic environment (Figure 3(b)). Natural hypoxic conditions inside EBs have been already demonstrated elsewhere [6]. In EBs lacking the *Hif1α* gene, endogenous *β*-catenin signalization was significantly lower and *Hif1α* knock-in returned luciferase activity back to the control level (Figure 3(c)). We also performed RT-qPCR analysis of genes from the TCF/LEF protein family, which are directly involved in the coactivation/repression of *β*-catenin downstream target genes (Figure 3(d)). We observed significant downregulation of *Lef1* in *Hif1α^−/−^* EBs, while the results did not show any substantial changes in *Tcf1*, *Tcf3*, or *Tcf4* gene expression. On the other hand, *Hif1α^−/−^* EBs manifested clear upregulation of the important proneural transcription factor *NeuroD1*. Taken together, these results suggest that stabilized HIF1*α* in EBs markedly contributed to active *β*-catenin signaling as well as to the suppression of the proneuronal cellular program in the first days of spontaneous differentiation.

### 3.4. HIF1*α* Attenuates the Neural Phenotype by the Stabilization of *β*-Catenin in EBs

**Figure 4:**
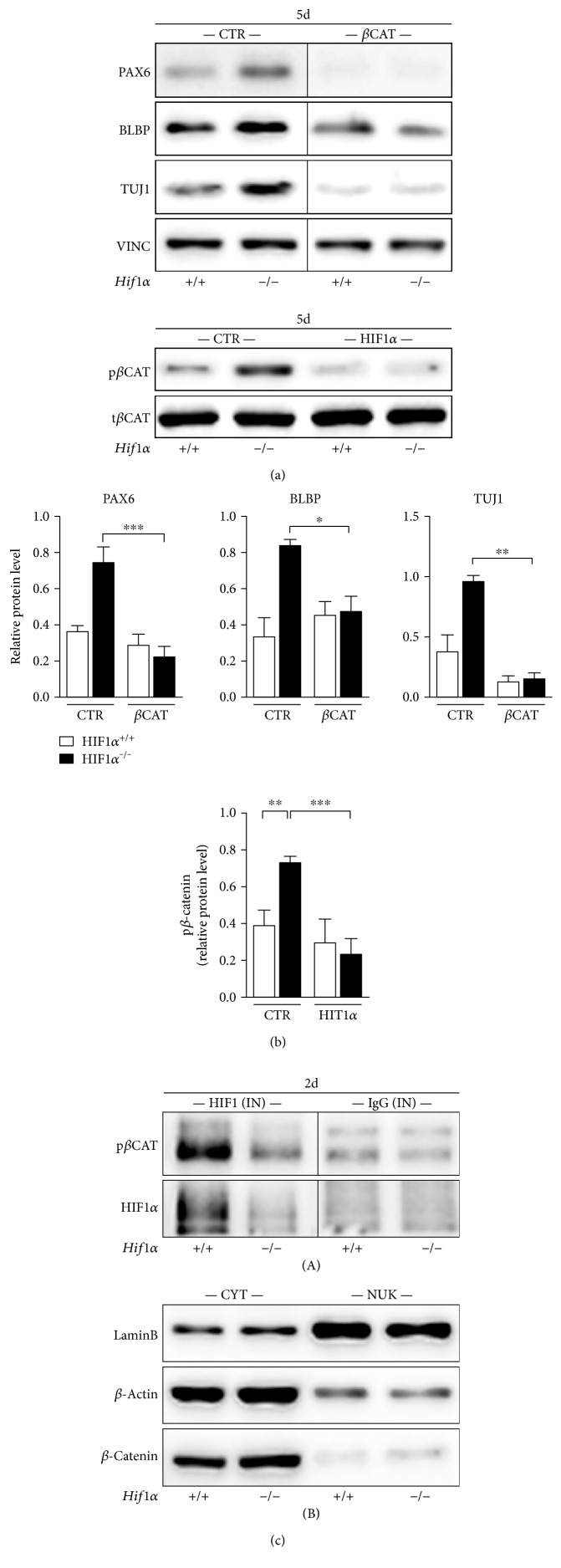
HIF1*α* attenuates neural phenotype by the stabilization of *β*-catenin in EBs. (a, A) The protein level of PAX6, BLBP, and TUJ1 in control (CTR) and *β*-catenin-transfected (*β*CAT) 5-day-old EBs (5d) derived from wild-type and *Hif1α*-deficient ESC. (a, B) Densitometric analysis and quantification (one-way ANOVA; *n* = 8 for PAX6, *n* = 4 for BLBP, and *n* = 3 for TUJ1). (b, A) Western blot analysis of control (CTR) and *Hif1α*-transfected (HIF1*α*) 5-day-old EBs showing level of phosphorylated *β*-catenin. (b, B) Densitometric analysis is based on at least 5 independent experiments (one-way ANOVA). (c, A) Immunoprecipitation analysis from nuclear fraction of 2-day-old EBs. The analysis was performed using either IgG (isotype control) or HIF1*α* antibody as an input (“bait”), and *β*-catenin was detected afterwards (“prey”) as well as HIF1*α* in the magnetic bead fraction. (c, B) LaminB and *β*-actin were used as loading controls for nuclear and cytoplasmic fractions, respectively. Cytoplasmic *β*-catenin level does not differ between the two lines. CTR versus *β*CAT samples (a, A) as well as HIF(IN) versus IgG(IN) samples (c, A) were loaded on the same gel, but not directly beside each other. All procedural conditions and settings, including exposure time, were the same for all samples.

Since previous results suggested the involvement of HIF1*α* in neural lineage shift as well as in the upstream regulation of *β*-catenin, we transfected growing EBs with plasmid containing construct for constitutive expression of *β*-catenin and analyzed the proteins involved in neural differentiation in 5-day-old EBs. We observed the overall suppression of neurogenesis in *Hif1α^−/−^* EBs after *β*-catenin ectopic overexpression (Figure 4(a)). PAX6 protein decreased nearly 3 times compared to nontransfected *Hif1α^−/−^* EBs. Also, BLBP protein decreased significantly in *Hif1α^−/−^* EBs after *β*-catenin transfection. The level of TUJ1, a clear indicator of immature neurons, decreased more than 5 times after *β*-catenin transfection and was, unlike BLBP and PAX6, noticeably lower than after HIF1*α* transfection, suggesting the relevant impact of *β*-catenin on neuronal fate. This also confirmed that *β*-catenin alone, in the absence of stabilized HIF1, is capable of attenuating the neurophenotype during spontaneous differentiation.

We then aimed to search deeper into the connection between HIF1*α* and *β*-catenin in spontaneous differentiation by addressing the degradation status of *β*-catenin in *Hif1α^−/−^* EBs. Surprisingly, we found that *Hif1α^−/−^* EBs had a markedly higher level of *β*-catenin phosphorylated on Ser33/37/Thr41, a modification which targets the protein for ubiquitination and thus degradation in the cytoplasm [21]. Importantly, the knock-in of *Hif1α* in *Hif1α^−/−^* EBs diminished the level of p*β*-catenin back to, and even below, the control level (Figure 4(b)). To check whether HIF1*α* directly binds to *β*-catenin, we performed immunoprecipitation analysis from the nuclear fraction of 2-day-old EBs, where stabilized HIF1*α* was already proven to be involved in *β*-catenin signaling (Figure 3). Nuclear fraction analysis showed the clear coprecipitation of HIF1*α* and *β*-catenin compared to the IgG input control (Figure 4(c), A) suggesting the direct interaction of both proteins. We also did not observe any differences in cytoplasmic *β*-catenin level after HIF1*α* knockout suggesting that the observed changes in neural phenotype are associated rather with nuclear *β*-catenin function (Figure 4(c), B). From these results, we conclude that stabilized HIF1*α* in cells within growing EBs is able to stabilize and bind to *β*-catenin, which results in enhanced nuclear *β*-catenin signaling during spontaneous differentiation.

## 4. Discussion

In this study, we present evidence that transcription factor HIF1*α* plays a fundamental role in neural fate during the spontaneous differentiation of ESC. We found that EBs derived from ESC with the ablated *Hif1α* gene underwent an abnormal developmental shift towards the neural lineage after ten days of differentiation in an adherent culture. Importantly, increased neural differentiation appeared in *Hif1α^−/−^* EBs after only 5 days of cultivation and the knock-in of *Hif1α* during EB formation returned the neural phenotype back to that observed in wild-type EBs. Research by Professor Simon's group demonstrated that EBs with a disrupted HIF1 complex exhibited inappropriate mesodermal and hemangioblast differentiation [5]. This was supported by a recent finding that EBs lacking the *Hif1α* gene exhibit the deregulation of important endodermal and mesodermal lineage markers and impaired cardiomyogenesis [13]. Lee et al. presented evidence that EBs can sustain a naturally hypoxic environment with upregulated markers of endoderm and mesoderm but not ectoderm; this phenotype was even strengthened when EBs were primed in additional hypoxia for two days and the knock-down of *Hif1α* abolished this lineage shift [6]. Overall, these results support our conclusions that HIF1*α* is a crucial transcription factor involved at the decision crossroad of ESC during spontaneous differentiation and that its stabilization leads to preferential endodermal/mesodermal fate while suppressing differentiation into neuroectoderm.

We did not observe the return of the abnormal neural phenotype back to normal physiological conditions after VEGF treatment of *Hif1α^−/−^* ESC during EB formation. VEGF was described as the main HIF1*α*-dependent effector responsible for vascular-lineage commitment in EBs primed in 1% hypoxia [6]. We assume that prolonged incubation in 1% hypoxia could artificially increase the stabilization of HIF1*α* and subsequently induce the strong expression of VEGF, which in turn could have a higher impact on vascular-lineage commitment, unlike our experimental conditions, which reflected the natural “semihypoxic” microenvironment of EBs.

In this study, we show a clear connection between HIF1*α* and *β*-catenin in the regulation of neural lineage blockade during EB maturation. We demonstrated that stabilized HIF1*α* in EBs naturally protected *β*-catenin from degradation and increased its nuclear activity which was associated with neural lineage fate blockade. Moreover, immunoblots clearly identified *β*-catenin-HIF1*α* protein interaction in the nuclear fraction of wt EBs. There is much evidence in developmental and cancer biology that HIF1*α* under hypoxic conditions coincides with the Wnt signaling pathway through *β*-catenin, which has substantial consequences for cellular fate and survival. In colorectal and hepatocellular carcinoma, HIF1*α* was found to compete with TCF-4 for direct binding to *β*-catenin enhancing transcriptional HIF1*α* activity [22, 23]. During muscle regeneration after ischemic injury, HIF1*α* negatively regulated skeletal myogenesis through the inhibition of canonical Wnt signaling [24]. However, the results of multiple developmental research rather point to the activation of canonical Wnt signaling after hypoxic HIF1a stabilization. iPS-derived embryonic bodies primed in hypoxia exhibited impaired maturation to cardiomyocytes; such EBs exhibited increased HIF1*α* signaling and Wnt canonical target expression and the colocalization of both HIF1*α* and *β*-catenin in the nuclear fraction [25]. In ESC subjected to hypoxic conditions, HIF1*α* was found to enhance Wnt/*β*-catenin signaling through binding to HRE elements in promoter regions of the *β*-catenin downstream effectors LEF-1 and TCF-1 [15]. Unlike, we here demonstrated that enhanced *β*-catenin signaling activity rather emerged from increased *β*-catenin nuclear translocation after its stabilization by HIF1*α* protein in the cytoplasm. These results indicate that the cellular outcome of *β*-catenin and HIF1*α* interaction is highly dependent on tissue type, developmental stage, and pathophysiological conditions, as well as oxygen level. One can even speculate that HIF1*α* seems to have an inhibitory effect on Wnt canonical signaling in carcinogenesis, whereas it rather supports *β*-catenin signaling and downstream targets during early embryogenesis and neurogenesis.

The role of *β*-catenin in the neural phenotype shift during EB differentiation was demonstrated by several of our results. The knock-out of the *Hif1α* gene preceded the induction of the neural phenotype as well as the downregulation of *β*-catenin signaling. Importantly, the transient increase in *β*-catenin expression was a sufficient signal for the inhibition of the neural differentiation in *Hif1α^−/−^* EBs. It appears that the impact of *β*-catenin overexpression on the reduction of the neuronal marker TUJ1 seems to be higher than after HIF1*α* reintroduction. These results rather favour *β*-catenin over HIF1*α* as the final regulatory effector in neural fate blockade. The involvement of *β*-catenin in neural fate blockade during ESC differentiation and development has already been described by several authors. The prevention of *β*-catenin signaling after the loss of the Wnt1/pLRP6 receptor or after the addition of the Wnt/*β*-catenin pathway inhibitor DKK1 resulted in increased neuroectodermal differentiation and a population positive for TUJ1 cells in differentiating ESC [26]. Treatment with soluble SFRP2 protein, an antagonist of canonical Wnt signaling, stimulated the expression of neural progenitor markers such as *Pax6* or *Ngn2*, as well as the emergence of cell immunoreactive for the TUJ1 protein during EB formation; treatment with Wnt1 ligand or GSK3 inhibitors inhibited this neuronal differentiation [8]. The focal elimination of *β*-catenin in neural precursors of the embryonic cortical ventricular zone *in vivo* caused premature neuronal differentiation [27]. Other research directly proved the connection between nuclear *β*-catenin and neural differentiation in both EBs and mouse development. EB formation from ESC with abrogated nuclear *β*-catenin signaling was accompanied by increased differentiation into neurons, as shown by an abundance of TUJ1-positive aggregates. Moreover, embryos originating from this ESC line were abnormally developed with increased markers of neural precursors and neurons such as PAX6 and N-CAM in the inner embryonic layer [28]. The induction of the neuronal phenotype in EBs with abrogated nuclear *β*-catenin is in line with our results where *Hif1α^−/−^* EBs with downregulated *β*-catenin signaling exhibited a marked increase in several neuronal markers during EB formation as well as later during adherent differentiation. We assume that, in our case, the observed effect of *β*-catenin on neural fate blockade during early spontaneous differentiation was rather due to its acting on an endogenous/physiological level and that the attenuation of *β*-catenin signaling in *Hif1α^−/−^* EBs led to increased neurogenesis.

Overall, our data clearly show that the stabilization of HIF1*α* results in blockade of proneural genes, including *NeuroD1*, as well as in the enhancement of *β*-catenin downstream effector *Lef1*. A similar regulation was discovered in hypoxic pancreatic *β*-cells, where the expression of the *NeuroD1* gene was suppressed by stabilized HIF1*α*, while the knockdown of *Hif1α* led to its upregulation [29]. Additionally, *β*-catenin may positively regulate HIF1*α* signaling by direct interaction, which was also documented elsewhere [22–24]. However, as already mentioned above, our data point to the possibility that *β*-catenin alone has the potential to regulate stem cell fate in EBs lacking HIF1*α*. The upregulation of transcription factor PAX6 in early neural progenitors is associated with the increased expression of important proneuronal genes, including *Ascl1* or *NeuroD1* [30]. Therefore, we consider the observed changes in the level of PAX6 as an early and crucial regulatory step determining subsequent neuronal fate in EBs. The upregulation of the transcription factor PAX6 after the downregulation of *β*-catenin was documented by several other authors [8, 28]. More importantly, the ectopic expression of *β*-catenin/LEF1 fusion protein in the cortical neural precursors of mouse embryo delayed the expression of several neurogenic genes, including PAX6, while the conditional ablation of *β*-catenin accelerated the expression of PAX6 [31]. Thus, activation of *Lef1* gene expression regulated by *β*-catenin seems to be the crucial upstream stimulus inhibiting the activation of the neuronal program in EBs. Still, the exact molecular mechanism underlying the neural inhibitory effect of HIF1*α* and/or *β*-catenin in EBs should be clarified by further research.

## 5. Conclusions

**Figure 5:**
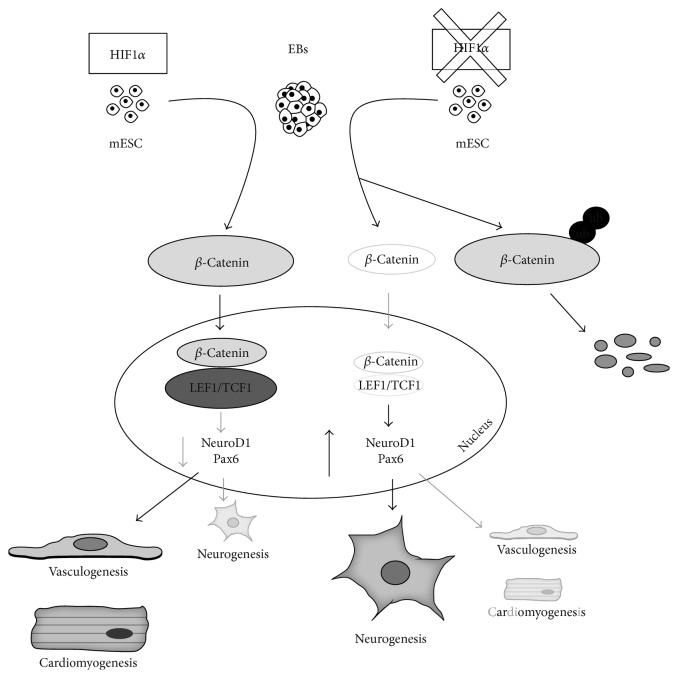
Scheme indicating the possible role of HIF-1*α*/*β*-catenin in the regulation of neurogenesis *in vitro*. During spontaneous differentiation, HIF1*α* is stabilized in the increasing hypoxic environment of growing EBs; this protects *β*-catenin from degradation in the cytoplasm, which in turn increases the localization of active *β*-catenin in the nucleus, where *β*-catenin activates several genes involved in mesodermal differentiation while inhibiting neural differentiation. In the absence of HIF1*α*, *β*-catenin is degraded at a higher rate, which reduces its activity in the nucleus, and the cell is driven to premature neurogenesis (increased *NeuroD1* and PAX6), probably through the downregulation of the *Lef1* gene (see Discussion for more detailed information and references).

In summary, we hereby demonstrate that the stabilization of HIF1*α* blocks the degradation of *β*-catenin in the cytoplasm and increases *β*-catenin nuclear activity, which results in the natural developmental blockade of neural differentiation in ESC (Figure 5). These results bring strong evidence that endogenous hypoxic niche within growing EBs has an essential impact on stem cell developmental fate during spontaneous differentiation. Since both HIF1*α* and *β*-catenin are fundamental factors in developmental as well as cancer biology, we believe that these *in vitro* findings can contribute to understanding the mechanisms of neural differentiation under both physiological and pathophysiological conditions.

## Supplementary Materials

Supplementary Materials Supplementary figures: (A) Immunohistochemical staining of (a) compact EBs colonies or (b) EBs resuspended to single cells revealed increased expression of TUJ1 in *Hif1α*^−/−^ after 10 days of differentiation in adherent culture. (B) Detailed image captured under light microscope showing *Hif1α*^−/−^ EBs differentiated for 10 days. There are several neurite projections sprouting from the colony indicating mature neurons. (C) Representative light microscope images showing 5 day-old EBs cultivated on agar. No apparent differences in size or morphology between *wt* and *Hif1α*^−/−^ EBs were observed. Scale bar: 200 μm. (D) Western blot analysis in EBs differentiated for 10 days in adherent culture. Recombinant VEGFα was added to the cells either during EBs formation (0-5d) or later during differentiation in adherent culture (6-13d). Neural stem cell marker LewisX and early neuronal marker TUJ1 were analysed. AKT was used as protein loading control.

## References

[1] Okazaki K., Maltepe E. (2006). Oxygen, epigenetics and stem cell fate. *Regenerative Medicine*.

[2] Quinn P., Harlow G. M. (1978). The effect of oxygen on the development of preimplantation mouse embryos in vitro. *Journal of Experimental Zoology*.

[3] Pabon J. E., Findley W. E., Gibbons W. E. (1989). The toxic effect of short exposures to the atmospheric oxygen concentration on early mouse embryonic development. *Fertility and Sterility*.

[4] Ying Q.-L., Wray J., Nichols J. (2008). The ground state of embryonic stem cell self-renewal. *Nature*.

[5] Ramírez-Bergeron D. L., Runge A., Dahl K. D. C., Fehling H. J., Keller G., Simon M. C. (2004). Hypoxia affects mesoderm and enhances hemangioblast specification during early development. *Development*.

[6] Lee S.-W., Jeong H.-K., Lee J.-Y. (2012). Hypoxic priming of mESCs accelerates vascular-lineage differentiation through HIF1-mediated inverse regulation of Oct4 and VEGF. *EMBO Molecular Medicine*.

[7] Yamaguchi T. (2001). Heads or tails: Wnts and anterior–posterior patterning. *Current Biology*.

[8] Aubert J., Dunstan H., Chambers I., Smith A. (2002). Functional gene screening in embryonic stem cells implicates Wnt antagonism in neural differentiation. *Nature Biotechnology*.

[9] Naito A. T., Shiojima I., Akazawa H. (2006). Developmental stage-specific biphasic roles of Wnt/*β*-catenin signaling in cardiomyogenesis and hematopoiesis. *PNAS*.

[10] ten Berge D., Koole W., Fuerer C., Fish M., Eroglu E., Nusse R. (2008). Wnt signaling mediates self-organization and axis formation in embryoid bodies. *Cell Stem Cell*.

[11] Majmundar A. J., Wong W. J., Simon M. C. (2010). Hypoxia-inducible factors and the response to hypoxic stress. *Molecular Cell*.

[12] Singh R. P., Franke K., Wielockx B. (2012). Hypoxia-mediated regulation of stem cell fate. *High Altitude Medicine & Biology*.

[13] Kudová J., Procházková J., Vašiček O. (2016). HIF-1alpha deficiency attenuates the cardiomyogenesis of mouse embryonic stem cells. *PLoS One*.

[14] Gustafsson M. V., Zheng X., Pereira T. (2005). Hypoxia requires notch signaling to maintain the undifferentiated cell state. *Developmental Cell*.

[15] Mazumdar J., O’Brien W. T., Johnson R. S. (2010). O2 regulates stem cells through Wnt/*β*-catenin signalling. *Nature Cell Biology*.

[16] Murry C. E., Keller G. (2008). Differentiation of embryonic stem cells to clinically relevant populations: lessons from embryonic development. *Cell*.

[17] Kotasová H., Procházková J., Pacherník J. (2014). Interaction of Notch and gp130 signaling in the maintenance of neural stem and progenitor cells. *Cellular and Molecular Neurobiology*.

[18] Roitbak T., Li L., Cunningham L. A. (2008). Neural stem/progenitor cells promote endothelial cell morphogenesis and protect endothelial cells against ischemia via HIF-1*α*-regulated VEGF signaling. *Journal of Cerebral Blood Flow and Metabolism*.

[19] Tomita S., Ueno M., Sakamoto M. (2003). Defective brain development in mice lacking the Hif-1*α* gene in neural. *Cell*.

[20] Veeman M. T., Slusarski D. C., Kaykas A., Louie S. H., Moon R. T. (2003). Zebrafish prickle, a modulator of noncanonical Wnt/Fz signaling, regulates gastrulation movements. *Current Biology*.

[21] Salic A., Lee E., Mayer L., Kirschner M. W. (2000). Control of *β*-catenin stability: reconstitution of the cytoplasmic steps of the Wnt pathway in Xenopus egg extracts. *Molecular Cell*.

[22] Kaidi A., Williams A. C., Paraskeva C. (2007). Interaction between *β*-catenin and HIF-1 promotes cellular adaptation to hypoxia. *Nature Cell Biology*.

[23] Zhang Q., Bai X., Chen W. (2013). Wnt/*β*-catenin signaling enhances hypoxia-induced epithelial-mesenchymal transition in hepatocellular carcinoma via crosstalk with hif-1*α* signaling. *Carcinogenesis*.

[24] Majmundar A. J., Lee D. S. M., Skuli N. (2015). HIF modulation of Wnt signaling regulates skeletal myogenesis in vivo. *Development*.

[25] Medley T. L., Furtado M., Lam N. T. (2013). Effect of oxygen on cardiac differentiation in mouse iPS cells: role of hypoxia inducible factor-1 and Wnt/beta-catenin signaling. *PLoS One*.

[26] Čajánek L., Ribeiro D., Liste I., Parish C. L., Bryja V., Arenas E. (2009). Wnt/*β*-catenin signaling blockade promotes neuronal induction and dopaminergic differentiation in embryonic stem cells. *Stem Cells*.

[27] Woodhead G. J., Mutch C. A., Olson E. C., Chenn A. (2006). Cell-autonomous β-catenin signaling regulates cortical precursor proliferation. *Journal of Neuroscience*.

[28] Rudloff S., Kemler R. (2012). Differential requirements for *β*-catenin during mouse development. *Development*.

[29] Sato Y., Inoue M., Yoshizawa T., Yamagata K. (2014). Moderate hypoxia induces *β*-cell dysfunction with HIF-1-independent gene expression changes. *PLoS One*.

[30] Sansom S. N., Griffiths D. S., Faedo A. (2009). The level of the transcription factor Pax6 is essential for controlling the balance between neural stem cell self-renewal and neurogenesis. *PLoS Genetics*.

[31] Machon O., Backman M., Machonova O. (2007). A dynamic gradient of Wnt signaling controls initiation of neurogenesis in the mammalian cortex and cellular specification in the hippocampus. *Developmental Biology*.

