# Supplementary figures and images for: Neural Differentiation Is Inhibited through HIF1α/β-Catenin Signaling in Embryoid Bodies
Source: Stem Cells Int. 2017 Dec 20;2017:8715798. doi: 10.1155/2017/8715798 (PMC5750467; doi:10.1155/2017/8715798)

# Supplementary figures

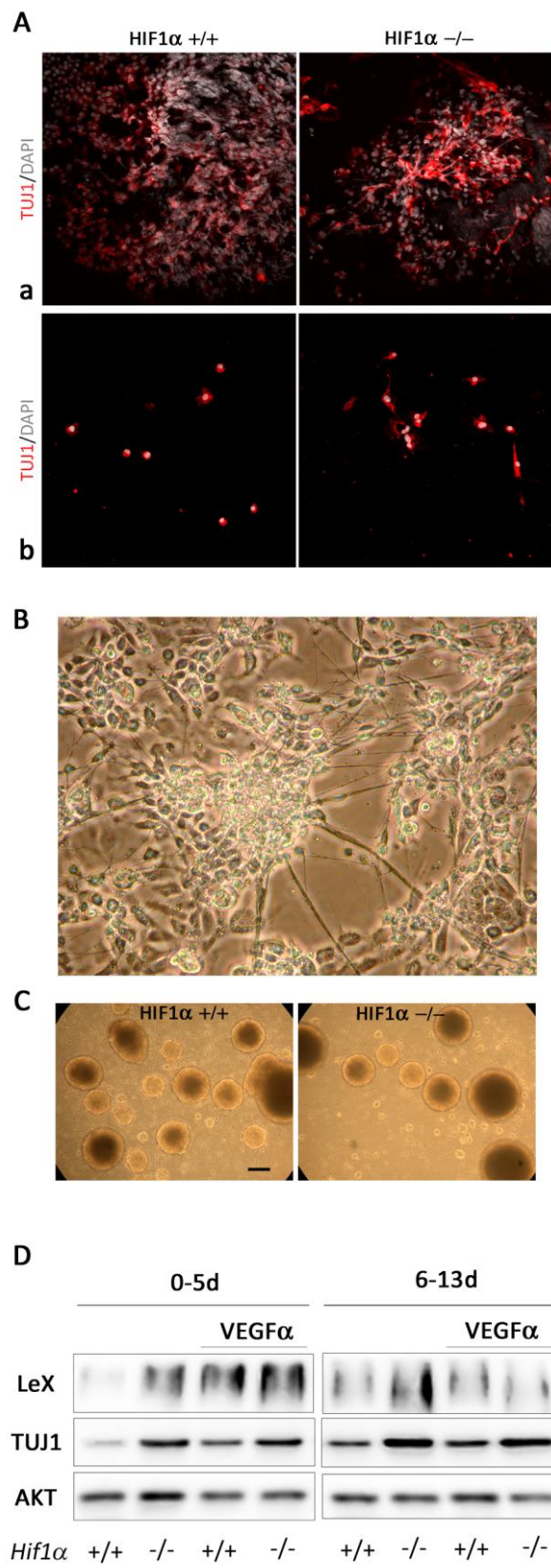

Supplement: Supplementary Materials — Supplementary figures: (A) Immunohistochemical staining of (a) compact EBs colonies or (b) EBs resuspended to single cells revealed increased expression of TUJ1 in Hif1α−/− after 10 days of differentiation in adherent culture. (B) Detailed image captured under light microscope showing Hif1α−/− EBs differentiated for 10 days. There are several neurite projections sprouting from the colony indicating mature neurons. (C) Representative light microscope images showing 5 day-old EBs cultivated on agar. No apparent differences in size or morphology between wt and Hif1α−/− EBs were observed. Scale bar: 200 μm. (D) Western blot analysis in EBs differentiated for 10 days in adherent culture. Recombinant VEGFα was added to the cells either during EBs formation (0-5d) or later during differentiation in adherent culture (6-13d). Neural stem cell marker LewisX and early neuronal marker TUJ1 were analysed. AKT was used as protein loading control. [file 8715798.f1.pdf]
